# Predicting and Synchronising Co-Speech Gestures for Enhancing Human–Robot Interactions Using Deep Learning Models

**DOI:** 10.3390/biomimetics10120835

**Published:** 2025-12-13

**Authors:** Enrique Fernández-Rodicio, Christian Dondrup, Javier Sevilla-Salcedo, Álvaro Castro-González, Miguel A. Salichs

**Affiliations:** 1 Department of Systems Engineering and Automation, University Carlos III of Madrid, Av. de la Universidad, 30, 28911 Leganés, Spain; 2 The Interaction Lab, School of Mathematical and Computer Sciences, Heriot-Watt University, Edinburgh EH14 4AS, Scotland, UK

**Keywords:** gesture prediction, deep learning, transformer models, co-speech gestures, human–robot interaction

## Abstract

In recent years, robots have started to be used in tasks involving human interaction. For this to be possible, humans must perceive robots as suitable interaction partners. This can be achieved by giving the robots an animate appearance. One of the methods that can be utilised to endow a robot with a lively appearance is giving it the ability to perform expressions on its own, that is, combining multimodal actions to convey information. However, this can become a challenge if the robot has to use gestures and speech simultaneously, as the non-verbal actions need to support the message communicated by the verbal component. In this manuscript, we present a system that, based on a robot’s utterances, predicts the corresponding gesture and synchronises it with the speech. A deep learning-based prediction model labels the robot’s speech with the types of expressions that should accompany it. Then, a rule-based synchronisation module connects different gestures to the correct parts of the speech. For this, we have tested two different approaches: (i) using a combination of recurrent neural networks and conditional random fields; and (ii) using transformer models. The results show that the proposed system can properly select co-speech gestures under the time constraints imposed by real-world interactions.

## 1. Introduction

Humans are compelled to create social relationships due to their need to be part of a group, even if the conditions for establishing such relationships are adverse [1]. The existence of a social bond between two persons can alter the way that one of them perceives information provided by the other [1]. This shows the benefits of establishing social relationships with the people who surround us. However, for the interactions required to forge this bond to happen, it is necessary for both interlocutors to recognise each other as acceptable partners for establishing a mental connection [2]. Based on this, it is reasonable to assume that for a robot to be able to bond with a person, it also has to be considered an acceptable partner by the person. This is particularly important in social robotics, where the main goal of the robot is to be able to interact with humans in accordance with a social model applied by the users [3]. The process of creating this bond with the users starts with giving the robot a lively appearance.

There is a large body of evidence in the field of robotics that supports the idea that an animate appearance is important for a robot that has to engage in a social role [4,5,6]. Gelman and Spelke [7] proposed that there are four features that are exhibited by animate beings and are not exhibited by inanimate ones. Out of the four, the two that roboticists tend to focus on are (i) giving the robot the ability to generate different intentions, desires, and beliefs (feature 3); and (ii) designing the expressions (in this work, we will use both ‘expression’ and ‘gesture’ interchangeably to refer to any coherent combination of multimodal information oriented to achieve a particular communicative goal) that the robot can perform (feature 1) [8,9,10]. In this work, we focus on the latter, taking the idea of animate entities being able to move on their own and extending it to give robots the ability to perform multimodal expressions that combine motions, light patterns, gazes, and the display of multimedia content (showing images or videos on a touch screen). However, while this task is easier to complete when the robot is silent, it becomes a harder challenge when the robot is simultaneously emitting a verbal message. The gestures used in these situations are known as co-speech gestures.

Co-speech gestures are non-verbal expressions that accompany verbal messages during face-to-face interactions. While the speaker with the initiative in the interaction uses these gestures to enhance the message being conveyed, the other participants in the interaction need to integrate both the verbal and non-verbal components of the message in order to fully understand what is being communicated [11]. Although some research suggests that the production of speech and the production of gestures are connected (for example, a person talking remotely to another person might still gesticulate even if the other participant in the conversation cannot see the gestures) [12], other researchers disagree and decouple the generation of these components [13]. According to the work of McNeill [14], the combination of speech and gestures can be viewed as integrating two contrasting semiotic systems with a common core idea, but with different ways of expressing it. In the same work, he also stated that although in these situations speech and gestures co-occur and share a common meaning, each component can also convey separate information. Co-speech gestures have attracted significant attention in the area of Human–Robot Interaction. On one hand, a natural interaction requires that the robot be capable of identifying and understanding the user’s expressions. Following this idea, Hegde et al. [15] proposed three tasks that can serve as a proxy for assessing applications of gesture recognition using the JEGAL model that matches gestures to words and phrases in the speech. Their approach aims at tackling the problems of sparse and weak cross-modal correlations and is capable of outperforming modern approaches like large vision-language models. Another example is the work of Lee et al. [16], where they used mobile robots for caretaker recognition, tracking, re-identification and gesture recognition using an LSTM-based model in a caregiving scenario. Their gesture recognition system presented an accuracy above 90% in real time. Ghaleb et al. [17] presented an approach to learning embeddings for representational gestures that leverages self-supervised learning techniques and semantic information from features extracted by large language models. While these works showcase the importance of understanding co-speech gestures, there is also an extensive body of work focused on the task of co-speech gesture generation.

Methods for endowing social robots with the ability to use co-speech gestures can be categorised into two main groups: (i) generating the expressions automatically; and (ii) handcrafting the expressions beforehand and synchronising them with the robot’s speech. In recent years, with the advent of machine learning models, the first approach (which we will refer to as gesture generation in this manuscript) has become the predominant one. The solutions in this category receive the robot’s speech (this can mean a transcription of the speech, the prosodic features of the speech, or both), along with other external and internal factors that might play a part in expression generation (for example, the affective state of the robot or the identity of the user), and create a sequence of non-verbal actions synchronised with this speech [18,19]. Usually, these types of systems tend to focus on body postures and motions. Using a machine learning model to dynamically generate all the expressions required enhances the variability of the robot’s expressiveness without forcing developers to handcraft a large library of gestures. However, expressions generated through this method can be more generic than those handcrafted by roboticists. Also, these methods tend to focus on generating expressions of one modality (with body motions being the most common modality) instead of generating multimodal expressions. Finally, gesture generation systems usually require large datasets to learn how to generate expressions. While these datasets are easier to compile when working with humanoid robots (as we can imitate human behaviour), this can become an issue when considering robots with non-humanoid forms or constrained expressiveness. In these cases, handcrafting the gestures the robot will use can ensure that they convey the desired message, given each robot’s configuration. When developing an expressiveness architecture that relies on a library of predefined gestures, there are two problems that we need to solve: (i) selecting the most appropriate expressions given the robot’s speech and other related factors; and (ii) synchronising the speech and the gestures. Traditionally, both problems were solved manually, which can be tedious and can lead to repetitive interactions. Relying on deep learning models can solve the issue of having to select which expressions should accompany the robot’s speech, and it can also help with the synchronisation task [20]. Automating the selection and synchronisation of expressions allows the developers of robotics applications to focus exclusively on the verbal dimension of interactions, simplifying their work. This approach, which we will refer to as *gesture prediction*, is the one that we have followed in this research.

In this work, we present a co-speech gesture prediction and synchronisation method for social robots. This method predicts the types of gestures that should accompany the robot’s speech, selects expressions of the correct type that fit the length of the speech chunk that they will be combined with, and finally computes the appropriate point in time when each gesture should be performed to achieve a proper synchronisation of the verbal and non-verbal components. The gesture prediction method receives the robot’s speech and generates a list of labels representing the types of gestures that should accompany this speech. Using labels to represent the types of gestures simplifies the use of multimodal gestures, as they can be designed beforehand, ensuring a proper combination of modes. Initially, we implemented a gesture prediction solution that combined long short-term memory (LSTM) neural networks, which are used to encode inputs, and conditional random fields (CRFs), which are used to generate the sequence of labels. More recently, we developed a new approach that takes advantage of a machine learning model that has fuelled significant growth in the area of natural language processing in the past few years: the *transformer* model.

Regardless of the prediction method used, the output is the same: a list of labels that indicate not only the types of gestures that should accompany the speech but also the point in the speech where each expression should start. Our synchronisation module receives the robot’s utterance divided into a list of tokens, along with the gesture type labels for each token, computes the lengths of the different speech chunks (fragments of speech that have been tagged with the same label) based on the number of characters in the text, selects appropriate expressions for each gesture label, and combines the speech and non-verbal gestures into a single expression that can be executed by the robot.

In summary, the contributions of this work are the following:

- The development of a gesture prediction method that receives the robot’s speech and generates a sequence of labels that represents the gesture/s that should accompany the speech.
- The development of a rule-based gesture synchronization system that connects speech and expressions and allows developers to define individual rules for each category of gestures.

The work described in this manuscript focuses on describing the implementation of the proposed solution and on validating this approach from a technical perspective. Because the proposed system will be integrated in a social robot that needs to be able to interact in a natural way, it is essential that the gesture prediction and synchronisation approach can complete its task at a speed that abides by the constraints imposed by real-time interactions. Also, it is important to optimise the use of resources, as computational power tends to be limited in robotic platforms. The subjective evaluation of the effect that a proper selection and synchronisation of co-speech gestures has over the perception that users have of the robot is outside the scope of this work.

The rest of the manuscript is structured as follows. Section 2 presents an analysis of related works and compares them with the solutions presented in this manuscript. The contributions of this paper are described in Section 3, which details the co-speech gesture prediction and synchronisation module. Section 4 presents a series of evaluations used to measure the performance of the gesture prediction model. The results obtained in these evaluations, along with the limitations of the proposed approach, are discussed in Section 5. Finally, Section 6 presents the conclusions extracted from this work and analyses the accomplished objectives.

## 2. Related Work

This section presents an analysis of the literature on both gesture generation (creating the expressions from scratch dynamically) and gesture prediction (selecting expressions from a library and connecting them with the robot’s speech), and these works are compared with the solution we present.

### 2.1. Gesture Generation

In recent years, a majority of the work done in the area of endowing an agent with expressiveness has focused on the automatic generation of expressions. A large portion of the solutions proposed have either focused directly on virtual agents or generated expressions for 3D skeletons that can later be adapted for virtual or physical agents. Speech-gesture synchronisation is achieved in most cases by generating expressions frame by frame. In 2019, Ginosar et al. [21] proposed using a convolutional network composed of an audio encoder and a 1D UNet translation architecture for generating gestures for an entire utterance that matches a particular gesticulation style. This ensures that the synthesised motions are smooth. The discriminator in this approach has to evaluate if the expressions generated match the required style. A large temporal context is used to overcome the asynchronicity between speech and gestures. The system proposed by Kucherenko et al. [22] uses a denoising autoencoder with a GRU-based encoder and decoder. The encoder converts the input speech into a low-dimensional representation, while the decoder converts this representation into a sequence of motions. Sadoughi and Busso [23] proposed the use of a dynamic Bayesian network for constraining gesture generation to the discourse function (e.g., if the speech is a question) and gesture prototypes (e.g., head nod). These constraints are represented by extra observational states in the baseline network. Once motions are generated, the proposed model smooths the trajectories between the key poses in the gesture.

A year later, Yoon et al. [18] proposed an architecture for gesture generation based on the user’s identity and the speech’s transcription and audio. The inputs are encoded separately, concatenated, and finally passed to a multilayered bi-directional GRU-based generator. To ensure a smooth transition between poses, the four previous poses generated are also passed to the model. Ferstl et al. [24] presented a system that passes the outputs of a generator network through four discriminators during training to ensure the realism, feasibility, non-repetitiveness, and smoothness of the expressions. To simplify the generation problem, they associate gesture phases with each time step of the dialogue. Also in 2020, Kucherenko et al. [25] presented an autoregressive model that uses FiLM conditioning so that the generation of motions is conditioned on previous poses and previous and successive speech features to add context. They use 10 and 20 frames of past and future speech, respectively. These time spans are taken from research in gesture-speech alignment. Ahuja et al. [26] presented a method that encodes the speaker’s gesticulation style and the frames of the agent’s speech using temporal convolutional neural networks (TCNNs) and passes these encodings to a 1D version of the U-Net generator, with TCNN-based sub-generators. The discriminator is also modelled as a TCNN.

In 2022, Yazidan et al. [27] proposed tackling the generation of co-speech gestures as a machine translation task. Their solution uses a denoising autoencoder to reduce the body pose dimensionality at the frame level, a vector-quantised variational autoencoder to cluster similar motion sequences into a single symbol within a codebook, and finally a GRU-based sequence-to-sequence machine translation model with a soft attention mechanism to translate speech into motion symbols. Chang et al. [28] proposed a similar approach, framing gesture generation as a sequence-to-sequence conversion task. Their approach generates expressions based on the user’s identity. For this, they used the Tacotron2 architecture [29] as a baseline and enhanced it with the addition of a locality-constraint attention mechanism that allows the decoder to learn the gesture-speech alignment from local audio features. Liang et al. [30] presented the *semantic energised generation* method for generating semantic-aware upper-body co-speech gestures. In this system, the *decoupled mining* module separates the semantic-irrelevant data from the speech data and passes both components through independent encoders, while the *semantic energised module* uses a decoder for gesture generation and a semantic prompter to encourage the networks to learn and generate semantic gestures.

More recently, in 2024, Bhattacharya et al. [31] developed a method for synthesising co-speech facial expressions and upper-body gestures for virtual avatars. Their GAN-based approach learns from the speech audio and transcript, the speaker’s ID and sparse 3D face landmarks and pose sequences from RGB video. Synchronisation between speech and expressions is achieved by generating the gestures as a temporal sequence of face landmark deltas and pose unit vectors. That same year, Qi et al. [31] proposed EmotionGesture, a framework for synthesising diverse emotional co-speech expressions. Their approach first extracts emotion and audio beat features from the speech. Then, it models the frame-wise alignment between the embedded beat features and the speech rhythm by using speech transcripts synchronised with frame-wise timestamps that align each word with a frame-wise gesture. Finally, it uses a Spatial-Temporal Prompter to generate temporally-coherent future expressions from a set of initial poses. Finally, a transformer model is used to generate 3D co-speech gestures. In 2025, Qi et al. [32] presented a co-speech generation framework aimed at building the interactions between concurrent gestures (gestures being performed by both parties in the interaction). For this, their approach establishes two cooperative transformer-based diffusion branches that generate gestures for two speakers through a denoising process. A Temporal Interaction Module is in charge of modelling the temporal association representation between each current speaker’s movements and conversational counterparts. Liu et al. [33] proposed GestureLSM, a flow-matching approach for co-speech gesture generation that models the interaction of different body regions through temporal and spatial attention. Their transformer-based approach divides the human body into different regions, and uses spatial attention to ensure coherence between body regions for each time step and then temporal attention to model motion progression.

Other authors have focused explicitly on developing gesture generation methods for robots. Aly and Tapus [34] presented an approach that takes a transcription of the user’s speech and uses it to infer the user’s level of introversion/extroversion, which affects the generation of expressions. Their system then combines two strategies for gesture generation. Metaphoric gestures are generated using coupled hidden Markov models (CHMMs), while the rest of the expressions are generated via a collection of XML-based modules that use rules for creating appropriate expressions and synchronising them with the speech. Alignment between gestures and words is achieved by using the TTS module to obtain the duration of the words and phonemes in the utterance, and using this information to schedule the beginning and end of each gesture. The method proposed by Ishi et al. [35] learns the conditional probability distribution that connects words to word concepts, concepts to gesture functions (e.g., iconic, metaphoric, etc.), and functions to gesture clusters. During generation, a gesture function is sampled from the probability distribution, a cluster is selected by taking into account the speaker’s identity, and finally, a random gesture is extracted (as long as the selected function is not *beat*). Beat gestures are generated based on prosodic features. The expressions can be discarded depending on the dialogue acts extracted from the speech. Gestures and speech are synchronised by connecting the stroke phase of the gesture with the beginning of the word it is connected to (if they are non-beat expressions) or to prosody peaks (for beat gestures). Yoon et al. [36] proposed using a GRU-based encoder-decoder architecture that uses the transcription of the robot’s speech split into chunks to generate 2D poses for the robot’s upper body joints. Their approach includes a soft attention mechanism that allows the decoder to focus on keywords in the sentence. Previous outputs are passed to the decoder to ensure smoothness. Yu and Tapus [37] presented a system that takes the audio signal and uses it to directly generate multiple expression sequences by adding random noise during the generation process. This seeks to give their system the ability to create multiple expression sequences for a particular utterance. A temporal GAN framework takes care of the alignment between the gestures and the speech.

### 2.2. Gesture Prediction

While most of the work done for endowing agents with the ability to use co-speech gestures focuses on automatic generation, there is still a large number of authors who have proposed approaches for automatically selecting appropriate non-verbal expressions. Among the authors that have focused on virtual agents, in 2014, Chiu and Marsella [38] proposed a machine learning-based method that uses CRFs for predicting if a gesture should be attached to the speech based on audio features of the speech, and then uses Gaussian process latent variable models to generate motions based on acoustic features and the gesture annotations. A year later, the same authors [20] presented a second method for mapping speech and gestures that builds on their previous work. This approach uses the joint learning of deep neural networks and a second-order linear chain temporal contingency method for mapping the temporal alignment between speech and gestures. The system computes the probability of each gesture label being the one that should be used, given an input sequence containing the speech’s transcription and prosodic features, and the part-of-speech (PoS) tags for the words in the speech. More recently, Kucherenko et al. [39] presented a framework for gesture generation that included a prediction stage in which the speech’s transcription and audio are fed into a convolutional neural network that predicts a set of binary gesture properties. These include the type of gesture and the gesture phase, among others. A normalising flow takes speech and gesture properties and describes a distribution from which motion sequences can be sampled. In 2024, Zhang et al. [40] used an end-to-end GPT-based model to generate gestures aligned with speech rhythm. Simultaneously, a Large Language Model tags the transcription of the speech with relevant semantic gestures. Finally, both outputs are combined. An alignment module is in charge of merging semantic and rhythmic gestures at the right timing. This module decides when and how to merge these gestures so that the naturalness of the original gesture is not affected. Gao et al. [41] presented GesGPT, an LLM-based approach to gesture generation. Their system first passes the speech through ChatGPT to generate a parsing script containing the intention of the message, the emphasis words, and the semantic words. Then, a second module uses this script to search for appropriate professional gestures. Finally, the selected gestures are fused with rhythmic base gestures.

Other authors have proposed approaches designed to be integrated into robots. Perez-Mayos et al. [42] presented in 2019 a combination of three methods for selecting and synchronising gestures based on speech features. The first one chooses gestures based on keywords found in the text, the second one selects them based on pitch peaks, and the last one combines the previous two methods, using both the text and the pitch as inputs. Speech-gesture synchronisation is achieved by extracting the time step for the item (keyword, pitch peaks…) that triggered each gesture. and using a timer that ensures that each gesture starts at the appropriate time. That same year, Ghosh et al. [43] presented a random forest-based model for selecting the most appropriate expression from a library of gestures. A year later, Xiao et al. [44] proposed a system for allowing robots to learn the correlation between speech and iconic and metaphoric gestures, based on examples taken from human-human interaction videos. Their method calculates dissimilarities between behaviours in a dataset, uses a regression network to predict dissimilarities between the input speech and the texts in the examples, and uses these dissimilarities to select appropriate behaviours. The gestures and the sentence are then sent to the robot’s animation engine to be executed side by side. A short delay is added before the beginning of the utterance to align the beginning of both modalities. Recently, Lim et al. [45] developed a co-speech gesture generation for interactions using American Sign Language system. User signs are analysed with ChatGPT, which then generates appropriate verbal responses and tags keywords in the robot’s speech with gesture labels.

### 2.3. Our Approach

The approach presented in this work seeks to predict which multimodal expressions are the most appropriate given the next utterance of the robot. Among the works reviewed in this section, the works presented by Chiu and Marsella [38], Chiu et al. [20], and Ghosh et al. [43] follow a similar approach. In particular, one of the solutions we present in this paper could be compared with the one presented by Chiu et al. in 2015, as both systems rely on a combination of deep neural networks with CRFs for predicting a sequence of gesture labels associated with the robot’s speech. There are two main differences between our work and the one proposed by Chiu et al. On the one hand, we opted to use the communicative intention of the robot’s speech as part of the information that the model can use for predicting the sequence of gesture labels. The model first identifies this intention from the utterance and then combines the speech and the identified intention to predict the labels. Because we consider that each sentence can have multiple gestures attached to it, even though it only has one intention, we believe that the use of two prediction steps can improve the results obtained with the system without forcing developers to pass any extra information to the model. The second difference is that our work adds a second prediction method, based on transformers, and compares its performance with the performance of the method based on the combination of RNNs and CRFs.

The use of the speech’s communicative intention is an approach that has been used in HRI previously. Other researchers like Sadoughi et al. [23] and Lugrin et al. [19] also considered similar features in their solutions. However, an important difference between these solutions and our work is that our system extracts the intention automatically from the robot’s speech, while these approaches require the intention to be provided as input. Additionally, while these other authors focused exclusively on the goal of the speech without context (e.g., ask a question or make a statement), our solution also takes into account the specific message that is being conveyed (for example, our solution distinguishes between a general question and a personal question about the other speaker). This makes it easier for the system to connect expressions that have been designed for more specific situations to the speech. The solution proposed by Ishi et al. [35] also considered the discourse function when generating the co-speech gestures. Their solution uses the discourse function to decide if the generated expression should be performed, but this function plays no role in selecting the expression itself. In our case, the intention predicted by the first model is fed into the gesture prediction model and affects the process of predicting the gestures.

## 3. The Co-Speech Gesture Prediction and Synchronization Module

In this section, we present our solution for predicting and synchronising gestures and speech based on the verbal message that the robot has to convey. While the prediction stage is platform-independent, the final steps in the synchronisation stage (creating a multimodal behaviour combining verbal and non-verbal information in a way that can be executed by the robot) will have to be adapted when the system is deployed on a new platform. The idea behind this system is to allow developers to design applications for robots by focusing exclusively on the verbal side of communication, simplifying their task. The non-verbal expressions that will be synchronised with the speech seek not only to enhance the robot’s animacy but also to help the verbal message to achieve its communicative goal.

**Figure 1:**
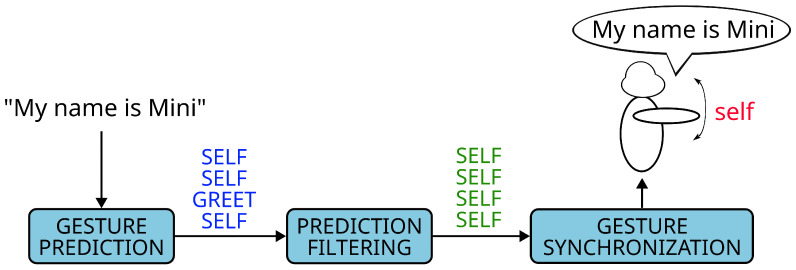
Pipeline for the proposed gesture prediction and synchronisation system. The labels in blue represent the sequence of gesture types generated by the prediction module, the labels in green represent the same sequence after the prediction errors have been filtered, and the label in red represents the specific expression that will be synchronised with the speech.

Relying on a closed set of expressions for combining speech and gestures requires two main tasks to be solved: (i) predicting the expression(s) that should accompany a given utterance; and (ii) synchronising both components in order to ensure that the timing between them is correct. Figure 1 shows the pipeline of the proposed co-speech gesture prediction and synchronisation module. Our solution performs three distinct steps:

1. Gesture prediction: The prediction stage receives the speech of the robot (this corresponds to the sentence ‘My name is Mini’ in Figure 1) and generates a list of labels that represent the types of expressions that should be associated with each part of the speech. In Figure 1, this corresponds to the sequence *SELF-SELF-GREET-SELF*, where *SELF* and *GREET* are two of the gesture types that we consider. Because each label is connected to a particular token, this also tells us at what point in the speech each gesture should be performed.
2. Prediction filtering: The list of labels generated during the prediction step is passed through a filter to ensure continuity between labels. Particularly, this filtering seeks to correct cases in which a random single label appears in the middle of a sequence of identical labels due to a prediction error. In the example shown in Figure 1, the label *GREET* was considered to be a prediction error and was replaced with the label *SELF*, matching the other three labels.
3. Gesture selection and synchronisation: The system selects one expression from the robot’s library for each sequence of consecutive identical labels (labels that represent the same gesture type) and synchronises it with the speech by computing the gesture’s starting point in seconds from the moment the speech starts. In Figure 1, we see that the expression *gesture_self* was the one selected from the expressions connected to the *SELF* label, and it was connected to the beginning of the robot’s speech, as shown by the arrow connecting these items.

### 3.1. Gesture Prediction

#### 3.1.1. Dataset

In this work, we have decided to frame the problem of gesture prediction as a *token classification* task, where an input text is first split into tokens, which are then labelled with a tag that indicates the class they belong to. While each token receives an individual label, the process is influenced by the entirety of the text. Two common examples of token classification that appear in the area of natural language processing (NLP) are PoS tagging, which involves assigning to each token a label that represents its syntactic function in the text (if it is a noun, adjective, verb, etc.), and named entity recognition (NER), which searches for known entities in a text and assigns them a label that indicates the type of entity they are (for example, a location, a person, or an organisation). We opted for modelling our problem this way because we consider that gesture prediction is a task that has to be performed at a sub-sentence level, that is, a single sentence could have attached multiple gestures in sequence. Dividing the sentence into tokens and classifying each of them separately can help to define the boundaries between gestures. Initially, we tackled the gesture prediction task using a combination of RNNs and CRFs. The process is divided into two steps: the first model extracts the communicative intentions of the robot’s speech, and the second model then generates the list of gesture labels. While we consider that each sentence has a single communicative intention, it can have multiple gesture types that are associated with it. Thus, the division of the gesture prediction task into two steps simplifies the process, but at the cost of increasing the latency. More recently, we decided to evaluate if more modern transformer models could outperform our original CRF-based approach. This new approach obtains the list of gesture labels directly from the transcription of the speech. Completing the task in one step (gestures from speech) removes intermediate tasks, simplifying the pipeline and reducing the latency introduced by the prediction system.

For training our prediction models, we decided to generate our own gesture prediction dataset. This gives us the freedom to choose the list of gesture types that we want our system to consider. The first step in creating the dataset was to define the list of gesture types and communicative intention labels that we wanted to use. The utterances used to create the datasets were extracted from the Cornell Movie Dialogs Corpus [46], a corpus that contains 304,713 utterances extracted from raw movie scripts. We have randomly drawn utterances from this corpus to create the instances for our dataset (we did not use all the utterances in the corpus due to its large size). The utterances in the corpus correspond to everyday informal conversations between people, which suits the task in which our robots will be used: acting as a companion for older adults, either in the user’s home or at daycare centres.

One of the features used during the gesture prediction process when using the CRF-based model is the communicative intention of the robot’s utterance. To compile the list of all the intentions considered, an annotator extracted a subset of utterances from the dataset and analysed them one by one. For each utterance, the annotator compared the intention that he perceived in it with the set of communicative intention labels already defined for previous utterances. If none of the existing labels fitted the intention perceived in the new utterance, the annotator would then add a new label to the set. This process resulted in a list of 28 different labels, including intentions like *greet* (the robot seeks to greet or say goodbye to a user), *state_user_fact* (the robot states a fact/opinion about the user), or *agree* (the robot shows agreement with the last statement made by the user), among others.

**Figure 2:**
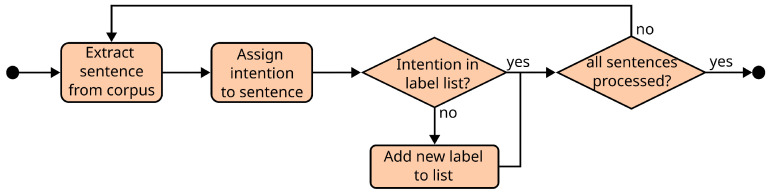
Flow diagram representing the process followed for obtaining the communicative intention labels.

**Figure 3:**
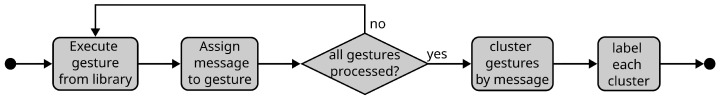
Flow diagram representing the process followed for obtaining the gesture labels.

While the communicative intention of the utterance is only considered by the CRF-based model, both solutions generate the same output: a sequence of gesture type labels. These labels were defined based on an analysis of all the multimodal expressions that the robotic platform on which we tested the system can use. An annotator observed the robot perform all the expressions one by one and wrote down the message perceived in each expression (for example, a gesture in which the robot raises and waves its hand with the palm facing the user can convey the idea of either greeting or saying goodbye to the user). Once the annotator observed all the expressions, those that conveyed similar messages were clustered together. Finally, a single label was defined for each group of expressions. The labels defined were chosen to define general gesture categories, as opposed to highly specific situations, with the goal that they can cover every situation that the robot might encounter. The final set of gesture types contained 21 different labels, including *question* (the robot asks a generic question), *self* (a reflexive expression in which the robot points at itself with the palm of the hand), or *sorry* (the robot shows remorse for something that happened and asks for forgiveness). The entire list of communicative intention and gesture type labels can be seen in Appendix A. Figure 2 and Figure 3 show the processes followed to obtain the lists of communicative intentions and gesture types, respectively.

We validated the library of expressions connected to the gesture labels to ensure that they convey the intended communicative messages. 15 participants who had prior experience with our robotic platform watched the robot perform each gesture alongside different utterances (all extracted from our dataset) and had to match each gesture to the utterance that matched it better. For each gesture, participants were presented with four possible utterances and were allowed to watch the robot perform the gesture alongside each utterance as many times as they needed. Next, they had to select the utterance that better matched the expression. They repeated this process for each gesture label. The results of the validation showed that 16 out of the 20 different expressions were correctly matched by a majority of participants. The four expressions that presented the worst recognition results were the ones for the labels *but*, *other_peer*, *question*, and *self*. These four expressions will have to be redesigned so they convey the intended message in a clearer way.

In total, the dataset generated contains 2600 instances. The average length of utterances in the dataset is 8.45 words, with the shortest having 1 word and the longest having 43 words. Because we used different frameworks for developing the two solutions proposed in this manuscript (CRF-based and transformer-based), we created two identical versions of this dataset. While the dataset used for training the CRF-based approach has each instance in a separate file, for the transformer-based we used the Dataset library provided by HuggingFace to structure our dataset. The latter version of our dataset is publicly available in HuggingFace (https://huggingface.co/qfrodicio, accessed on 12 November 2025). Listing 1 shows the structure of one of the instances in the dataset used to train the CRF-based approach. The example only shows the first token extracted from the utterance, along with its corresponding communicative intention and gesture type labels. The complete instance can be seen in Appendix B. Each instance contains an utterance that can include one or more sentences, the list of tokens into which this utterance is split (where each token contains the word, lemma, and PoS tag), the communicative intention(s) associated with the utterance (each token is labelled with a communicative intention label), and the list of gesture types that should be predicted for that utterance. While each sentence in the utterance tends to be labelled with a unique intention, gestures can be attached only to a specific part of a sentence, so a sentence can be labelled with a sequence of gestures. Communicative intention and gesture labels are defined using the IOB format, which uses prefixes to indicate if a label is the beginning (B-), inside (I-), or outside(O-) of an entity (a group of identical labels). In our particular case, all the tokens in the utterance belong to an entity, so the O- prefix will not be used.

**Figure 4:**
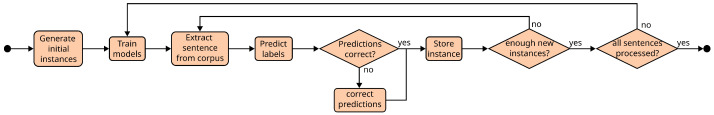
Flow diagram representing the process followed for generating our dataset.

The instances in the dataset were generated through an iterative process. First, a small subset of instances was handcrafted and used to train a preliminary version of the CRF-based model. Then, the trained models were deployed on a server. An annotator extracted utterances from the corpus one by one and passed them through the prediction models to generate the lists of communicative intentions and gesture types. The annotator manually reviewed the output of the models and corrected any label that, in his opinion, was incorrect. Finally, the utterance, the tokens, the communicative intentions, and the gesture types were stored in a file, and the process was repeated for a new utterance. Every 300 instances, the model was retrained with all the instances generated up to that point. This strategy was used to speed up and simplify the process of generating the dataset, as correcting a wrong prediction requires less time and effort than creating the instance from scratch. Also, the number of corrections that instances require diminishes as the dataset grows and is used to improve the performance of the model. The dataset generation process can be seen in Figure 4. To train the CRF-based and transformer-based models, the instances were split into training, validation, and testing subsets, with a 60-20-20 distribution, and it was ensured that the labels that the models need to predict were represented accordingly in each subset (e.g., 60% of the instances used in the training subset contain 60% of all the appearances of each label in all instances).
**Listing 1**. Schematic example of an instance in the dataset.      <?xml version="1.0" encoding="UTF-8"?>              <example id="1573945815700">              <sentence>                             Well, for starters, what do you do?              </sentence>              <tokens>                             <token id="1"                                            lemma="well"                                            pos="INTJ"                                            surface="Well"                             />                              …              </tokens>              <intentions>                             <token id="1"                                            value="B-REQUEST_PERSONAL_INFO"                             />                              …              </intentions>              <gestures>                             <token id="1" value="B-NEUTRAL" />                              …              </gestures>              </example>


#### 3.1.2. CRF-Based Prediction Module

The original approach that we developed was the CRF-based solution. We chose this particular model due to the results that CRFs have achieved in token classification tasks, including in gesture prediction [20,38]. This makes this approach a solid baseline to compare the performance of the transformer models with. The CRF-based prediction module is divided into two separate models. The first one takes the tokenised utterance and predicts the communicative intention behind it, and the second one takes both the tokenised text and the predicted intention and predicts the list of gestures that should accompany the speech. Both models follow the same hybrid approach based on a combination of RNNs and CRFs. The former gives the model the ability to learn dependencies between all elements in the sequence of tokens that represent the robot’s speech, as well as their PoS and communicative intention labels. The latter, on the other hand, gives the model the ability to take into account dependencies that might exist between the labels generated by the model (either the communicative intention of the speech or the predicted gesture types) based on features present in the input of the CRF (in our case, these inputs are the encodings generated by the neural networks). This combination of RNNs and CRFs has already been tested in labelling problems such as NER [47], dialogue act tagging [48], and keyphrase extraction [49].

Because the CRF-based model requires the inputs to be a list of tokens and PoS labels, we need to add a pre-processing stage. The first step is removing any special characters in the text, except for the delimiters (as they indicate separations between sentences and thus possible boundaries between communicative intentions and gesture types). Then, the text is passed through a PoS tagger provided by the spaCy library (https://spacy.io/, accessed on 12 November 2025). This transforms the sentence into a sequence of tokens, each containing a word, its lemma, and its PoS labels. To improve the performance of the PoS labelling stage, contractions are transformed, so that adverbs or contracted verbs are separated (e.g., *don’t* is transformed into *do n’t*).

We have used the AllenNLP library [50] to develop the prediction models used in our co-speech gesture prediction methods. This library has been developed using PyTorch (version 1.13), and it is designed for developing deep learning models for NLP tasks. The abstractions provided by AllenNLP make it possible to modularise the design of the models and create configuration files for training and evaluating these models. This library also provides a series of methods for solving common NLP tasks.

**Figure 5:**
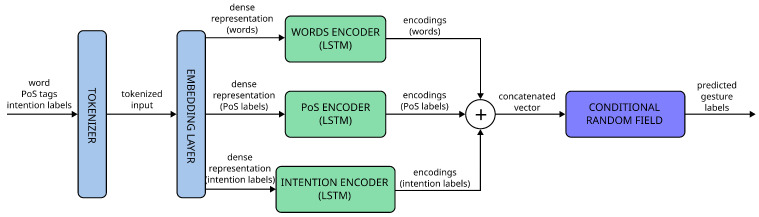
Architecture of the proposed model. This figure represents the version of the model used for predicting the gesture semantic values. The model for predicting intentions uses the same structure, but without the LSTM encoder for the intention labels.

The architecture of one of our prediction models can be seen in Figure 5. We have developed two models with similar architectures. The first one receives the robot’s speech and the sequence of PoS labels associated with it, and generates a sequence of labels that represent the speech’s communicative intention. The second model receives the same inputs as the first one, but it also receives the labels predicted by the first model; it generates a second sequence of labels representing the type of gesture that should be performed alongside the speech. In both cases, the same steps are followed after the model receives its inputs:

1. First, the model tokenises each of the inputs and represents each of these tokens with an ID.
2. Dense representations in a vector space are obtained for each ID by passing it through an embedding layer. We use a combination of the basic embedder provided by AllenNLP and ELMo [51] for obtaining these representations. The advantage of ELMo is that it considers not only the word itself but also the context in which it is used. We pass the inputs through each of the two embedders and then concatenate the representations generated.
3. The output of the embedding layer for each type of input (words, PoS labels, and, in the case of the second model, intention labels) is passed through an independent encoder, modelled as a stacked bi-directional LSTM network (two LSTM networks connected sequentially, so that the output of the first one is used as the input of the second one).
4. The outputs of the encoders are concatenated into a single vector and then sent to the linear-chain CRF model.
5. The CRF uses the Viterbi algorithm [52] to find the sequence of labels that have the highest probabilities based on the inputs of the model.

#### 3.1.3. Transformer-Based Prediction Model

**Figure 6:**
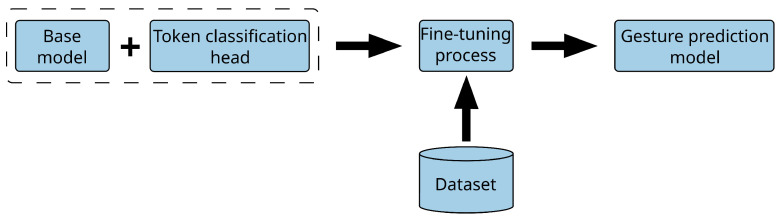
Process followed for fine-tuning transformer models for the problem of predicting co-speech gestures.

The development of new deep learning models and training techniques that improve performance while optimising the training process has resulted in significant growth in the area of NLU in the last few years. One of the models that has contributed the most to these advances is the *transformer* model [53]. This is a deep learning architecture based on the concept of self-attention, that is, learning to focus on certain elements in the input data depending on their importance. While there have been other models that incorporated attention mechanisms, transformers rely on attention alone, which has allowed them to outperform these other approaches. As mentioned before, we have framed the gesture prediction problem as a token classification task. This allowed us to take transformer models pretrained for this task and fine-tune them with our own dataset for gesture prediction to see if they could outperform our original method. To do this, we took three transformer models pre-trained for a general task, replaced the head of these models with a new one for the task of token classification, and then fine-tuned them with our dataset for gesture prediction. This process is shown in Figure 6.

One of the transformer models that has been widely used in multiple NLP tasks is the bidirectional encoder representations from transformers (BERT) model [54]. BERT is designed as a multi-layer bidirectional encoder that uses either a single text or a pair of texts as input representations. In this case, text can refer to one or multiple contiguous sentences. While traditional language models only consider the context that either precedes or follows the section of text that is being evaluated, BERT is bidirectional, which means that the context from both sides is considered for each text section. To achieve this, two pre-training objectives are used simultaneously: (i) for the *masked language modelling* objective, the model is asked to predict masked words in an input sentence; and (ii) for *next sentence prediction*, the model is asked to predict if two concatenated sentences were contiguous in the original text. Thanks to its bidirectional nature, BERT can be fine-tuned for different tasks by adding an extra output layer. Initially, BERT achieved state-of-the-art results for 11 NLP tasks, including those under the General Language Understanding Evaluation (GLUE) benchmark, the Stanford Question Answering Dataset (v1.1 and v2.0), and the Situations With AFiguredversarial Generations (SWAG) dataset. Because of the performance achieved, it has since become the baseline in many NLP works. Due to its widespread use, we have decided to test its applicability to the task of gesture prediction.

When selecting which implementation of BERT to use, we decided to evaluate the correlation that might exist between a model’s size and its performance for the task at hand. Thus, in addition to fine-tuning the base implementation of BERT, we have also selected a smaller model and a bigger model (in this case, size refers to the number of parameters of the model). We selected DistilBERT as the smaller model and RoBERTa as the bigger model. DistilBERT [55] was born from the desire to develop a model that could come close to BERT’s performance while also being smaller and faster, so that it could be integrated into systems with more constrained computational capabilities. RoBERTa (robustly optimised BERT pre-training approach) is an optimisation of BERT that seeks to correct the significant under-training of the original model [56].

The pre-trained implementations of all three BERT-based models used in this work were obtained from HuggingFace, a platform for sharing machine learning models and datasets. Among the different features it offers, the one that has been used for this work is the Transformers library, a package that simplifies the process of downloading pre-trained models from repositories, fine-tuning them, and generating inferences. The dataset and the fine-tuned models developed in this work are available on HuggingFace (https://huggingface.co/qfrodicio, accessed on 12 November 2025). We have used the same dataset described in Section 3.1.1, but with two modifications. First, the CFR-based model that the dataset was originally compiled for received the intention and gesture labels directly as strings, while the models we selected from HuggingFace expect integer values. Thus, it was necessary to create a mapping from strings to integers and apply it to the dataset. This mapping process has to be undone to convert the outputs of the model back into a list of string labels. The second modification was related to the tokenisation process. While the CRF-based model received the input text already converted into a list of tokens, the transformer-based models receive the utterance directly and then tokenise it with their own tokeniser. This can result in situations in which the token list generated by these new tokenisers and the list of tokens in the dataset instance do not align. In these cases, we need to apply a correction to the corresponding list of gesture labels to ensure that the number of output labels matches the number of tokens and that the label distribution of the original instance is maintained. For example, if a word is considered a single token in the dataset but is split into two tokens by the new tokeniser, we need the gesture label associated with the original token to be used to tag both of the tokens generated by the tokeniser.

#### 3.1.4. Training the Gesture Prediction Approaches

For training both approaches, we determined the most appropriate hyperparameters using Optuna [57], an automatic hyperparameter optimisation framework. Table 1 presents the complete list of hyperparameters used for the transformer-based and CRF-based approaches, along with the maximum and minimum values defined for each during the automatic search.

In all experiments, our goal was to identify the best-performing configuration for each model while maintaining a fair and practically deployable comparison between models on our robotic platform. We therefore used Optuna to run the same automated search procedure for all architectures over the hyperparameters listed in Table 1. The ranges for learning rate, weight decay, and batch size were centred around values commonly used for sequence labelling with LSTM–CRF and transformer models. Preliminary runs showed that values much larger led to unstable training, whereas values much smaller mostly slowed down convergence without improving validation performance. For the CRF-based models, we also optimised the size of the task-specific embedding layer, the hidden-layer size and number of recurrent layers, the dropout probability, the gradient norm clipping threshold, and the early stopping patience. Here, the bounds were set to cover a spectrum from compact to moderately large architectures that can still be run in real time on the Mini robot, rather than very large models that would be difficult to deploy on-board. For each model family, Optuna evaluated 20 trials and selected the configuration that maximised the F1 score on the validation split; the resulting settings are reported in Table 2.

Among these hyperparameters, *Embedding layer size* indicates the number of nodes in the embedding layer used in combination with ELMo in the CRF-based approach, *Hidden layer size* and *Number of recurrent layers* indicate the number of nodes in the first layer and the number of LSTM networks connected sequentially, respectively, in the LSTM-based encoders, *dropout* indicates the probability for the dropout layer introduced after every layer on the LSTM-based encoders (except for the last one) during training to reduce overfitting, *weight decay* indicates the decay that is applied to the weights in all layers of the model (except for bias and normalization layers) during training, *patience* refers to the number of epochs the trainer will wait before early stopping the training if no improvement is observed, and *gradient norm* indicates the maximum value the computed gradient norms will be scaled to.

For the transformer-based approach, we fine-tuned versions of the BERT base (https://huggingface.co/bert-base-cased, accessed on 12 November 2025), RoBERTa (https://huggingface.co/roberta-base, accessed on 12 November 2025), and DistilBERT models (https://huggingface.co/distilbert-base-cased, accessed on 12 November 2025), which were downloaded from HuggingFace. The same Optuna procedure and search space for learning rate, weight decay, and batch size were applied to each of these models, and the best configurations according to the validation F1 score are also summarised in Table 2. The number of epochs was chosen to monitor the evolution of the training metrics; in all three cases, we kept the model from the epoch with the lowest validation loss, which occurred at epoch 4.

### 3.2. Smoothing the Output of the Prediction Models

Regardless of the prediction model used, the output of the previous step is always the same: a sequence of labels. This can be a problem, as mistakes made during the prediction stage for communicative intentions could change the way that gestures are selected and synchronised, hindering the robot’s expressiveness. For example, if the robot wants to utter the sentence *‘Hello, how are you?’*, the gesture that should be performed alongside this sentence should be a *greeting* gesture. However, if, due to a prediction mistake, the sequence of gesture type labels ends up having, for example, a *thanking* gesture type label in the middle, this would lead to the module trying to synchronise three gestures: a *greeting* gesture, a *thanking* gesture, and finally a second *greeting* gesture. In order to mitigate this problem, we have implemented a filter for smoothing the predictions of the model. The filter is based on two main ideas: (i) identical labels tend to be together in the sequence; and (ii) a label will never appear alone in a sequence. Based on these ideas, the filter does the following:

1. First, the filter stores the positions in the sequence in which each label appears.
2. If a label appears only in consecutive positions, we consider that those positions form a *closed cluster*.
3. For any label that does not form a *closed cluster*, the filter splits the sequence of positions in which it appears into subsequences of consecutive positions. If any of these subsequences is larger than a threshold, then it is also labelled as a *closed cluster*. Otherwise, it is considered an *open cluster*.
4. We consider that the labels for the positions in *closed clusters* have been correctly predicted, and as such, they are stored in a list *L*.
5. The filter then selects the largest *open cluster A*, defined by its lowest and highest positions ($A_{l}$ and $A_{h}$, respectively). If none of the correctly predicted labels in *L* fall between $A_{l}$ and $A_{h}$, then all the positions between $A_{l}$ and $A_{h}$ are filled with the label from *A*, and the cluster is stored in *L*.
6. The previous step is repeated until none of the remaining *open clusters* can fit into *L*.
7. The clusters in *L* are ordered based on their lowest position, in ascending order.
8. Finally, if any position *i* in the label sequence is not present in *L*, then it is assigned the label from i−1.

Because the CRF-based prediction module includes two separate models (one for predicting communicative intentions and another for predicting expressions), we apply this filtering step to the output of both models, as a mislabelled communicative intention label in the middle of the sequence can also affect the quality of the prediction provided by the gesture prediction model. For the transformer-based model, we only need to apply the filter to the sequence of gesture labels.

### 3.3. Gesture Selection and Synchronisation

Finally, once we have our sequence of gesture labels and it has been passed through the filter to ensure continuity, the last step in the process is selecting the appropriate gestures from the robot’s library and connecting them to the appropriate points of the speech. Our robots rely on a library of handcrafted multimodal expressions that have been created for different tasks and situations. Each of the gesture labels that we have defined is associated with one or multiple expressions of different lengths. This will allow our system to synchronise appropriate gestures with sentences of different lengths.

**Figure 7:**
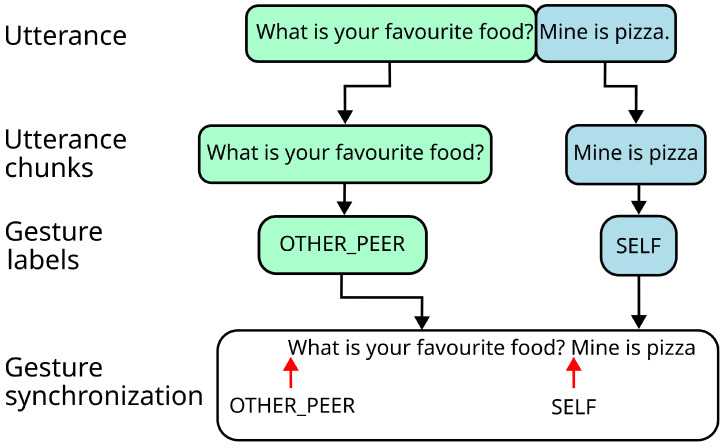
Example of how gestures are synchronised with one of the utterances in the case of use. The arrows in red represent the connection between the beginning of a gesture and a specific point in the speech.

In this work, we synchronise the verbal and non-verbal components based on the length of the robot’s speech using a rule-based method. Developers can create multiple rules for each gesture type and order them based on priority. These rules can specify three synchronization approaches: (i) attach the beggining of the gesture to a fixed position in the speech chunk (beginning, middle, or end), or to the appearance of a specific word or PoS label (e.g., attach the beginning of a negative gesture to the appearance of the word *no*, or to the appearance of the first adverb). Using the list of gesture type labels obtained from the prediction phase, the utterance is split into speech chunks, where each chunk corresponds to a segment of the utterance labelled with the same gesture type. The synchronisation process will be performed independently for each chunk. For example, if the module receives the utterance *‘What is your favourite food? Mine is pizza’*, the prediction stage would label the first question with the *other peer* gesture type (a gesture in which the robot points at the user in a non-threatening manner) and the second statement with the *self* gesture type (a reflexive gesture in which the robot looks at its own shoulder). Then, the utterance would be divided into two chunks: *‘What is your favourite food?’* and *‘Mine is pizza’*. This example can be seen in Figure 7.

For each of the speech chunks, the next step is computing the length in seconds. For this, the system retrieves the rules associated with the gesture to be synchronised, and checks them one by one. If the rule specifies the appearance of a word or PoS label, the synchronisation method searches the chunk for this item and, if it appears, computes the time length from the item to the end of the chunk. If it has to be attached to a specific point in the chunk, then it computes the duration from that point to the end of the chunk. Because the TTS integrated in our platforms does not provide information regarding the length of the utterance or other timing information, we decided to calculate the utterances’ duration empirically, based on the number of characters in them. To do this, we first remove all special characters in the text (including separators), take the number of remaining characters in the chunk, and multiply it by a conversion factor in order to obtain its duration in seconds. This conversion factor has been calculated experimentally for the TTS used in our robotic platforms. Once the duration of the speech chunk has been computed, the synchronisation module extracts all the expressions connected to the gesture label the chunk was tagged with, discards those that cannot be performed in the amount of time required for uttering the speech chunk, and selects one of the remaining gestures randomly. If all of the expressions associated with a given label are too long or no rule can be applied (e.g., the required word or PoS label does not appear in the speech chunk), then no gesture is synchronised with that particular chunk. Our solution does not modify expressions so they fit a particular utterance due to the gestures being multimodal and having a specific communicative meaning. We consider that changing aspects of the expression could create a mismatch between the different modalities, which could in turn result in the gesture losing its meaning.

If an expression has been selected, the synchronisation module computes its starting point. First, the synchronisation module takes the durations of every chunk preceding the one that is being processed and sums them. This gives us the starting point of the chunk in seconds from the moment that the robot starts to utter the entire speech. If the rule used specifies a synchronisation point different from the beginning of the chunk, the system adds to the starting point that was just calculated the duration of the fragment of the chunk before the expression’s starting point. The process of finding the duration of the chunk, selecting an appropriate expression (if possible), and computing the chunk’s starting point will be repeated for every speech chunk. Finally, the synchronisation module generates a single message containing the speech and the list of expressions, along with their starting points, and sends it to the robot’s expressiveness management module to be executed.

## 4. Evaluation

To test the capabilities of the proposed co-speech gesture prediction and synchronisation system, we present two different sets of experimental results: (i) the results of the objective metrics measured during the training/fine-tuning and evaluation of the gesture prediction modules presented in Section 3.1; and (ii) an analysis of the resources used by the models (GPU and GPU memory) and the time required to predict and synchronise gestures for the robot’s speech.

### 4.1. The Social Robot Mini

**Figure 8:**
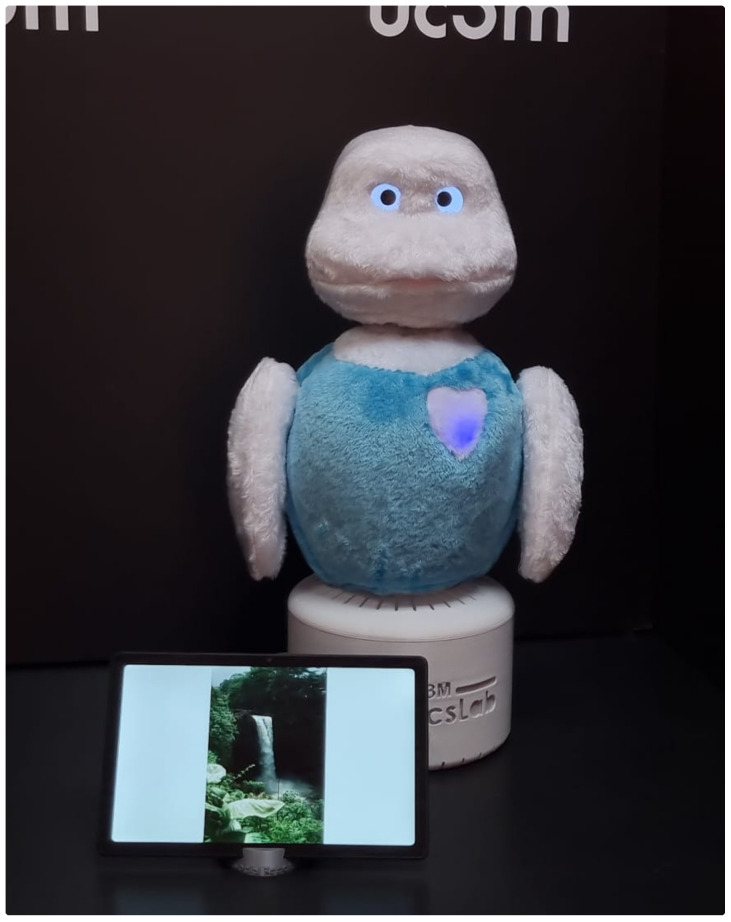
Mini, a social robot developed for interacting with older adults that suffer from mild cases of cognitive impairment.

The proposed gesture prediction approach has been integrated in Mini [58], a social robot designed with the goal of providing 24/7 assistance to older adults who suffer from mild cases of cognitive impairment. Mini, shown in Figure 8, is designed as a tabletop robot for one-to-one interactions. Its expressiveness capabilities include five joints (one per shoulder, two in the neck, and one in the base), an LED heart that can light up in different colours, two OLED screens used for displaying the robot’s eyes, integrated speakers for uttering speech and other non-verbal sounds, and a touch screen that is used for both displaying multimedia content and interacting with the user via menus. It is important to mention that, while Mini has been designed to interact with older adults, the proposed gesture prediction and synchronisation architecture was developed to be used in general-purpose robots, and thus the training of the models was not tailored to suit any particular demographic. Regarding its computational power, Mini is equipped with an Intel i5-3550 CPU with four cores running at 3.3 GHz and 16 GB of RAM. Mini’s software architecture runs on Ubuntu 16.04 (64-bit).

Mini’s hardware has a limitation for deploying deep learning models, as it lacks a dedicated GPU and does not have enough computational power for running these types of models with a performance that matches the temporal constraints imposed by real-world interactions. In order to overcome this limitation, Mini can connect through a socket-based connection to an external server equipped with an Intel Core i9-10900K CPU running at 3.7 GHz, two NVIDIA GeForce RTX 3090 GPUs, and 64 GB of RAM. When launching the gesture prediction and synchronisation architecture, we can choose to deploy it in the robot or in the external server. For the latter, Mini sends the utterance that needs to be passed through the model, and receives the gestures predicted, with the points in time at which each of them has to start. The following YouTube video (https://youtu.be/OxBceJ-G3CI, accessed on 12 December 2025) shows an example of how the proposed system works in a real task with Mini. In the video, Mini and a user play a quiz game (the video shows only one question for the sake of brevity).

### 4.2. Performance of the Prediction Models Used

We decided to evaluate all models using the multi-label classification metrics provided by the Scikit-learn library [59]. In particular, we opted for computing the weighted averages for the precision, recall, and F-score as a way to compensate for any imbalance in the presence of the different labels in the dataset. For this, the metrics function first computes these metrics for each class (gesture/intention label) independently. Then, the results obtained are weighted by the number of appearances of the class in the instance. Finally, the metrics function computes the average value for each of the three metrics. We selected this metric over seqeval [60] (the metric traditionally used for evaluating models trained for token classification) because it allows for partial matching of sequences. For example, if the model predicts the sequence of labels *AAABB* for an instance for which the correct sequence is *AABBB*, seqeval would return an F-score, precision, and recall of 0 (the same as if the model had predicted all wrong labels). Allowing for partial matching makes it so that the result of the evaluation reflects how close the predicted and correct sequences are.

After the models were trained, they were evaluated using the test split of the datasets, which contains 423 instances. To conduct this evaluation, we deployed the different models, passed each utterance in the test split of the dataset through them, compared the predicted sequences of gesture type labels with the correct labels defined in each instance from the test split using the metrics described above, and computed the average values for the full test split. These results are shown in Table 3.

If we compare the results obtained, we see that, while the differences in F-score between the transformer models are under 0.01, there is a higher difference between these models and the CRF-based approach (f-score of 0.744). For the other metrics, RoBERTa shows the highest precision (0.797), DistilBERT showed the highest accuracy and F-score (0.7827 and 0.7804, respectively), and BERT showed the highest recall (0.7874).

We also evaluated the effect that the filtering stage had on the predicted sequences of labels. While the transformer-based approaches have a single filtering stage after the gesture prediction is completed, the CRF-based approach filters both the communicative intentions and the gesture labels. After comparing the results, the variation in F-score is negligible (around 0.01 in all cases except for the DistilBERT-based approach, for which the difference is 0.0033).

**Figure 9:**
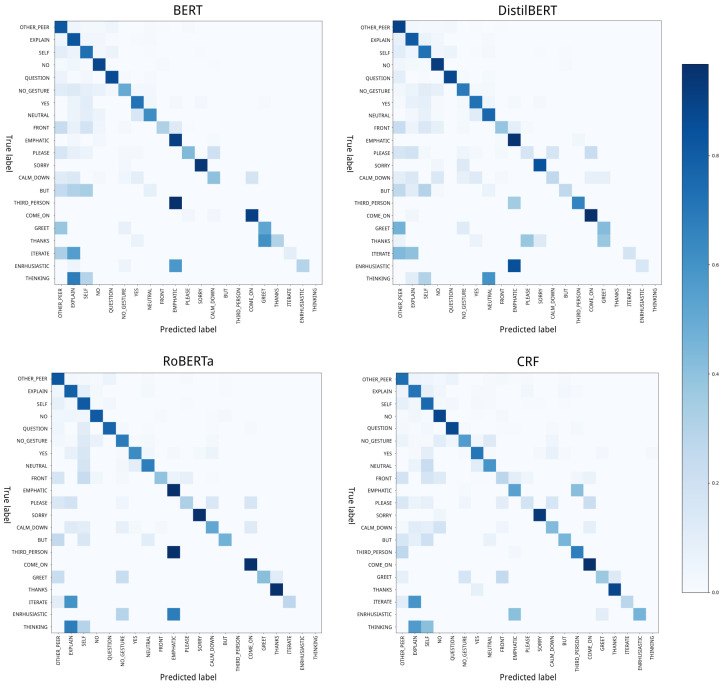
Confusion matrices showing the performance per label of the different models. For the sake of readability, the complete matrices with the data per cell can be found in Appendix C.

Finally, we also extracted confusion matrices for all approaches, showing the results per label. These matrices are shown in Figure 9. For the sake of readability, the complete matrices with the data per cell can be found in Appendix C. In these matrices, the rows have been ordered from the most frequent label in the dataset to the least frequent, meaning that *other_peer* (the label in the first row) is the gesture type that appears in the most instances, while *thinking* is the one that appears in the least. When observing the results, we see that both the BERT-based and DistilBERT-based models show consistently high prediction success for labels with higher presence in the dataset (for all top 10 labels, the correct option was predicted the majority of times), the success rate decreases for labels with less presence (for the bottom 11 labels, the BERT-based model only predicted the correct label the majority of times for 5 labels, while the DistilBERT-based model did it for 4). For the RoBERTa-based and CRF-based approaches, we see that the results are more consistent, with the RoBERTa-based model predicting the correct label the majority of times for 7 out of the 11 least present labels, and the CRF-based approach doing the same for 8 labels.

### 4.3. Resource Usage and Task-Completion Time

After evaluating the level of performance that the different models selected could achieve for the task of gesture prediction, we measured the system’s use of resources and the time required to complete the task (execution time from now on). For this evaluation, we prepared two different experimental setups. In our first test, the gesture prediction module was deployed on an external server, while the utterances used to evaluate the models were deployed in Mini. For the second evaluation, we deployed the prediction models directly in the robot to evaluate if it would be possible to dispense with the external server. This would allow our system to be used in situations in which the communication between the robot and external machines (like our server) is unreliable or simply not possible (for example, if the robot is placed at a user’s home in an area with a bad network connection).

For this test, we extracted the utterances from 213 instances contained in the test split of our dataset and sent them to our prediction and synchronisation module one by one. These utterances have an average length of 39.47 characters (with a standard deviation of 30.52), with the shortest texts having only 7 characters and the longest having 229. This is relevant because it affects the inference time. The instances of the dataset are loaded one by one into Mini and then sent to the prediction and synchronisation module (which would be deployed either on Mini or on the external server). For each utterance, our system predicts the gesture labels that best suit it, synchronises the appropriate gestures with the utterance using the rule-based approach, and returns the resulting multimodal expression. For the evaluation in which the models were deployed remotely, Mini was connected to the internet via Wi-Fi throughout the entire evaluation, while the server was connected through an Ethernet connection. To measure the system’s resource usage, we took different measurements depending on where the models were deployed. When deploying the models on the external server, we were able to take advantage of the computational capabilities that GPUs provide when dealing with deep learning models. Thus, during the evaluation, we measured the GPU computing time usage (understood as the percentage of time that at least one of the GPU’s cores performs operations) and the GPU memory used every 0.5 s, starting from the moment the evaluation began (when our system loaded the first utterance) and continuing until the last utterance was returned with its corresponding gestures; then, the average value was calculated. On the other hand, Mini is not equipped with a GPU, and the models had to be deployed on a CPU. Because of this, during this second evaluation, we measured the CPU’s computational load and the amount of RAM used. In both evaluations, we measured the system’s task-completion time (the time required to send a sentence to the server, predict and synchronise appropriate gestures, and return the result to the robot) as the average value of the time required to predict and synchronise gestures for each of the utterances in our test dataset.

When we analyse the results obtained when the models were deployed on the external server (Table 4), we see that the results are positive for all transformer-based approaches when it comes to the GPU computing time consumed by the models, as they all consumed between 1–2% of the available GPU computing time (understood as the percentage of time that at least one of the GPU’s cores performs operations). This percentage strongly increased for the CRF-based approach, which required 23.1% of the GPU computing time. If we focus on the memory requirements, all the models show similar performances, ranging from 5.2531% of the RAM consumed by DistilBERT (the lightest of the models tested) to 9.11% of the RAM consumed by the CRF-based approach. As expected, DistilBERT shows the best overall results (as it is the smallest model), requiring only 1.15% and 5.25% of the GPU computing time and RAM, respectively. It is followed by RoBERTa (2.024% and 6.2392%, respectively) and BERT (2.1951% and 6.001%, respectively). However, the large deviation indicates that all three transformer-based models performed similarly.

If we focus on the results obtained when the models were deployed locally in Mini (Table 4), we can see that the results are still positive in terms of the use of RAM but not in terms of the computation power required to run the models. We see that the results follow the same trend observed when the models were deployed remotely, with the CRF-based approach requiring a larger amount of resources and the transformer-based models showing more similar results. If we analyse the use of the CPU’s computing power, we see that the CRF-based approach shows the worst performance, requiring 560.5% of the computational power provided by one of the CPU’s cores (this means that the model would use 5.5 of the available cores). Among the transformer-based models, DistilBERT again showed the best overall results, requiring 148.28% of the computational power provided by one of the CPU’s cores and 5.54% of the available RAM, compared to the requirements of BERT (205.67% and 6.92%, respectively) and RoBERTa (222.92% and 7.42%, respectively). While in the previous test, the high deviation indicated that all transformer-based models performed similarly, here, the low deviation of the measurements does indicate a difference between the models’ performances.

Regarding the evaluation of the execution times, we had to define an appropriate threshold that would allow us to classify the performance of our system as either acceptable or not acceptable. During interactions with users, introducing a long delay in the robot’s responses can affect how users perceive the robot. A common rule used in human–robot interaction (HRI) is the *‘two seconds’ rule* [61], which states that the system’s response to a user’s input should be delivered in less than 2 s for the response to be perceived as natural. While this rule was originally proposed for human-computer interactions, researchers like [62] have tested its applicability to HRI. Other researchers like [63] observed that users preferred robots that introduced a small delay into their responses and that the preferred response times were around 1 s or less. This is why, in this work, we have decided to consider both thresholds: the robot should be able to respond to the user in 1 s, and in any case, this time should never be higher than 2 s.

**Figure 10:**
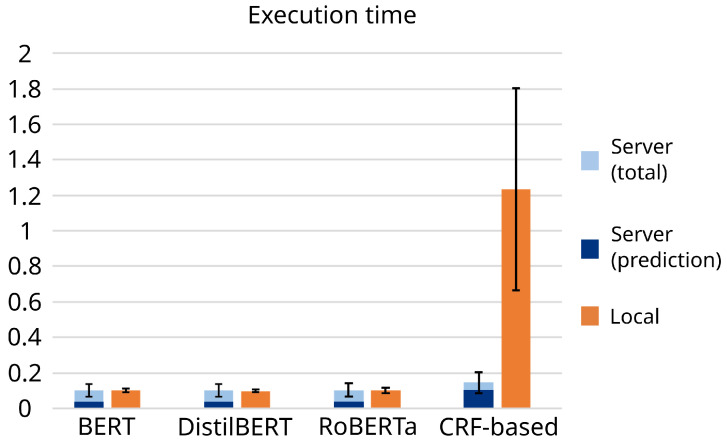
Execution times for all four models, when deployed locally and on the remote server. For the latter, the dark blue bars represent only the time for obtaining the prediction, while the light blue bars also include the time required for the robot-server communication. The bars represent average time values, while the whiskers represent the standard deviation.

During the evaluation in which the models were deployed remotely, we measured two time intervals for each utterance: (i) the time that passed between the moment Mini sent the utterance to the server and the moment it received the resulting multimodal expression (the light blue bar in Figure 10); and (ii) the time that passed between the moment that the prediction and synchronisation module received the utterance from the robot and the moment that it generated the multimodal expression (the dark blue bar in Figure 10). This allows us to evaluate the average time that our system requires for generating a response, as well as the delay introduced by the communication between Mini and the server. When the models are deployed locally, the delay due to communications disappears, and thus we measured only the time that passed between the moment each utterance is sent to the prediction and synchronisation module and the moment that a response is received (this would equate to the first of the two time intervals described above). The results are shown in Figure 10.

If we compare the results obtained, we see once again that the transformer-based models outperform the CRF-based approach. Among the transformer-based models, the one that shows the best results is DistilBERT, which is able to predict appropriate expressions and synchronise them with the speech in 0.0362 s. BERT and RoBERTa showed results very close to these results (0.0385 and 0.0387 s, respectively). The time required by the model based on CRFs was significantly higher (0.103 s). The results also show that the time required by our system to predict and synchronise gestures is significantly smaller than the delay introduced by the communication between the robot and the server. This makes the differences in performance between the models almost negligible, except in the case of the CRF-based approach. Additionally, while on average all approaches were shown to be fast enough to comply with the requirements that we have defined (the *two seconds* rule) without any issues, there were cases in which problems with the network connection caused the total time to spike, although it always stayed below 1 s (around 0.9 s in the worst cases observed).

When comparing the results obtained when the models were deployed locally and remotely, we see that for the transformer-based models, the results are similar in both cases. However, this is not true for the CRF-based approach, as the time required to predict and synchronise non-verbal gestures based on the robot’s speech increases to 1.23 s. This can be explained by the fact that our model uses 5 LSTM networks for encoding the different inputs used when predicting gestures. While the prediction time is below the two-second threshold that we have defined, in practice, it would leave a very tight time window in which the rest of the robot’s architecture would have to convey the generated multimodal expression to the user, almost ensuring that the two-second threshold would not be met.

## 5. Discussion

The results of the evaluations presented in Section 4 have led us to draw several ideas about the proposed system. The evaluation of the models showed that the new transformer-based approaches outperformed our original CRF-based solution. This falls under our expectations, considering the improvement in performance that transformer models have shown in multiple tasks in NLP when compared with other approaches. The fact that BERT and DistilBERT show similar results could be expected, as the original authors of the paper in which DistilBERT was presented [55] reported that this model was able to retain 97% of BERT’s performance on several tasks. However, we found it surprising that these two approaches performed at the same level as RoBERTa, an optimised version of BERT. When adding the filtering stage (two stages in the case of the CRF-based approach), we observed that there was little variation in the results. This also falls under our expectations, as the filtering stage simply smooths the label sequences, which could indistinctly result in a mispredicted label being corrected, or in a properly predicted label being replaced with the wrong one to ensure continuity in the sequence with the previous and following labels.

The transformer models show a second advantage, which is their lower resource usage and faster task-completion time compared to the CRF-based module. This agrees with our expectations, as the CRF-based module consists of two models running sequentially, with five total LSTM-based encoders and two CRFs. This was particularly clear when the models were deployed directly in Mini, as the amount of available resources is more constrained. In these situations, the CRF-based approach was not able to match the desired performance in terms of the time required to generate the predictions or the amount of resources used. The transformer-based approaches, on the other hand, were able to perform at an acceptable speed, meeting the threshold defined (the *two seconds* rule), but they still consumed more resources than desired (1.5 full cores of the CPU had to be exclusively dedicated to running DistilBERT, the best-performing model). While deploying the models on an external server specifically designed to run machine learning models solves the resource usage problem, it introduces other issues. In particular, we observed that there were instances when the communication between the robot and the server would slow down due to network problems. Although these cases were rare, this is still an issue that needs to be taken into account, particularly when the robot is deployed in environments where a stable network connection is not guaranteed. Regardless, our results show that the proposed system is able to work at a speed suitable for real-world interactions the majority of the time. On the other hand, centralising the deployment of these modules on an external server can lead to scalability issues if the number of robots attempting to use the proposed architecture is too large. Overall, the results seem to suggest that the fine-tuned version of DistilBERT is the best option, giving the same level of performance at a smaller cost.

If we analyse the performance of the different models depending on the gestures that had to be predicted, we saw that all models performed the best when predicting gestures that are more common in the dataset, while showing worse results when faced with less common gestures. However, there are exceptions to this, as the *come_on* and *sorry* labels showed great recognition rates for all models, while the *thanks* label also showed good results when using the CRF-based and the RoBERTa-based solutions. There is one fact that needs to be mentioned when analysing these results: in the gesture prediction task, an objectively wrong prediction would not necessarily hinder the interaction. For example, if the robot wants to ask a personal question about the user, the predicted gesture should be *other_peer* (the robot extends the arm towards the user in a non-threatening way, inviting them to answer). Instead, if the model labels the utterance with the *question* label (an expression for when the robot wants to ask a generic question), this would be an incorrect prediction. However, we believe that the selected gesture type could still be perceived as natural by the users, given the context of the situation (asking a question), and thus, this prediction error might not have a negative effect on the user’s experience. For example, this situation can be observed in the results for the *third_person* label when using the BERT-based and RoBERTa-based models. This type of gesture is considered to be associated with situations where the robot is discussing facts about a third party not present in the conversation in an aggressive manner (equivalent to a person extending the arm perpendicularly to the direction in which the other peer of the conversation is standing). Both models tended to label utterances that should be accompanied by this type of gesture with the *emphatic* label, which corresponds to a generic expression where the robot gesticulates in an aggressive manner, intended to be used in heated conversations about a generic topic. It could be argued that, while the models made a mistake, the gesture that they ended selected could also fit the utterance, as both gestures are thought to be used during an aggressive discussion. Another result that is worth mentioning is the case of the *enthusiastic* gesture type. We observed that all models tended to tag utterances containing this label with the *emphatic* label instead. Both gesture types involve the robot performing energetic motions, but *enthusiastic* expressions should be associated with more positive utterances (for example, the robot talking about something that it likes). A possible reason for this is that, without the context of the conversation and other speech features like the intonation, the models could be paying too much attention to factors like how the utterances are constructed, or the presence of exclamation marks. It also plays a role in the fact that *enthusiastic* is the second least common label in the dataset. After analysing all the results obtained with our original CRF-based solution and the new transformer-based approach, we can conclude that the latter is the optimal choice, and will be the one that will be integrated in our robotic platforms.

Finally, while the scope of this work focuses on the technical aspects of the development and integration of a co-speech prediction and synchronisation module on a social robot, we have also studied the effect that such an approach would have on how users perceive a robot capable of using appropriate co-speech gestures. In the work described in [64], we conducted a within-subjects evaluation where participants interacted with two robots: one that used co-speech gestures that match the content of its speech, and another that used generic, neutral expressions. The context for the evaluation was an interaction where first the participants observed in person an argument between a researcher and the two robots, and then they were asked to play a card game with the robots. The entire interaction was scripted and, for the robot that used the proper co-speech gestures, the robot’s speech was passed offline through the co-speech prediction module. All predicted expressions were synchronised to the beginning of their corresponding speech chunks. After completing the interaction with the robots, participants had to complete a questionnaire where they evaluated the overall appearance of the robot in terms of *sociability*, *agency*, *animacy* and *disturbance*, the *naturalness*, *semantic coherence* and *expressiveness* of the robot’s gestures, and general questions about which robot performed better during the game. The results showed that the robot that used the predicted co-speech gestures was perceived as having a higher level of agency, and its gestures were perceived as being more expressive. These results, while not explicitly validating the performance of our co-speech prediction and synchronisation module, do point towards the importance that such a system can have during human–robot interactions.

### Limitations

The proposed gesture prediction architecture has proven to be usable in real situations, as shown in the video included in Section 3. However, there are still some limitations of the work presented in this manuscript that would have to be addressed in future works. First, when compiling the dataset used for training the models presented in this paper, a single annotator was in charge of assigning gesture types and communicative intentions to the utterances in the corpus. This introduces a bias in the dataset and could alter how people perceive our robot when using the gesture prediction approach that we have proposed. The main objective behind this work is to develop a module able to automatically select the most appropriate expressions for a social robot from a gesture library based on the robot’s speech. Because of this, we have focused on presenting objective measurements that demonstrate whether the system can be used in embodied agents during real interactions. In this case, we consider that any possible bias introduced by having a single annotator should not impact the results of these evaluations, as assessing how users perceive these expressions is outside of the scope of this work. Regardless, in future works, the dataset needs to be validated through a crowdsourced process, where multiple participants assess the validity of the original annotations. For the communicative intentions, participants can be presented with the utterances in the dataset and asked to select the intention label that better suits the content of the speech. We can then compare the selections made by participants with our original labels. For validating the gesture labels, the process would involve showing participants the robot saying each utterance while it performs the gestures, and asking them to rate how natural the whole expression was.

A second issue that needs to be mentioned is the process that we followed for creating the dataset. Because we extracted sentences randomly from the corpus to create our dataset instances, this led to an imbalance in the number of appearances of each label, with those used in more common situations (asking a general question, for example) appearing more than those used in very specific situations (for example, a situation where the robot simulates to be thinking about something). As an initial step to understanding the effect that this imbalance has on the performance of the proposed model, we created two other versions of our dataset. For the first one, we removed all gesture labels that appeared in fewer than 100 instances, while for the second one, we removed those that appeared in fewer than 300 instances. For this, we took the original dataset and removed any instance that was labelled with one or more of the discarded gesture classes. With the former approach, we were left with 9 gesture labels and 2195 instances, and with the latter, we kept 5 gesture labels and 1748 instances. We used these modified datasets to finetune the three transformer models and compared the results to the ones obtained with the original dataset. This process resulted in an increase in *F1* score of between 1% and 4% for the dataset with 9 classes, and between 5% and 8% for the dataset with 5 classes. This shows that a proper rebalancing of the dataset could enhance the performance of the model significantly. In order to achieve this, in future works, the dataset needs to be extended by adding more instances for the underrepresented classes. This can be achieved by using data augmentation techniques that generate new instances similar, but not equal to, the ones already present in the dataset. A second potential solution that we will evaluate in future work is using our prediction models to identify all utterances in the corpus that could be labelled with one of the underrepresented classes, and then validate each one manually. Also, switching to a few-shot learning approach could remove the requirement for rebalancing the dataset, although it could also potentially introduce higher latencies and present other limitations that would have to be dealt with. Regardless, it would be interesting to test this approach and see how it performs compared to our current solution. Additionally, currently we have a limited number of expressions for each gesture label. Thus, it would be beneficial to expand the gesture library. These new expressions will have to be validated later through a user study to ensure that they convey the appropriate communicative messages. One last limitation of the method proposed is related to the synchronisation process. If the gesture’s length is larger than the speech chunk, then the gesture is directly discarded. Having a method for dynamically adapting the length of the expressions so that they fit the chunk they are associated with could enhance the performance of the proposed solution. This should be done while maintaining the integrity of the communicative message conveyed by the expression.

Regardless of these limitations, the performance of the co-speech gesture pipeline has met the expectations that we had at the beginning of this work, which has led us to consider the development of this module as a success.

## 6. Conclusions

In this paper, we have presented a machine learning-based architecture for predicting the most appropriate non-verbal expressions that a social robot should perform while uttering a given verbal message and synchronising both components. We have tackled the gesture prediction task as a labelling problem, where the model receives the sentences that the robot has to utter and predicts a set of labels that represent the gesture semantic values that should be connected to each word. For this stage, we proposed two different approaches. The first one used a combination of LSTM networks and CRFs and divided the prediction task into two steps: (i) predicting the communicative intention of the robot’s verbal message based on the words in the utterance and their part-of-speech labels; and (ii) using the words, PoS labels, and predicted communicative intentions to select the types of gestures that should accompany the speech. The second approach sought to simplify this pipeline by using transformer models, which eliminated the need to divide the problem into two steps; the transformer models are able to predict the appropriate gesture types from the words in the utterance alone. In both cases, we tried to improve the robustness of the proposed pipeline by adding filtering steps that try to correct potential prediction errors that could lead to discontinuities in the gesture label sequence generated by the models.

To synchronise the speech and gestures, our approach takes the list of gesture labels generated by the prediction stage, uses it to divide the robot’s utterance into speech chunks, and connects each gesture to the beginning of the corresponding chunk. The prediction and synchronisation stages are completely independent (as long as the output of the prediction stage is a sequence of gesture type labels), which allows us to modify or replace either stage without affecting the other, simplifying the process of testing new approaches for solving either task that might appear in the future. The proposed system has been integrated into a real robot, as shown in the video presented in Section 4 in which one of our robots played a quiz game with a user. Along with this example, we have also presented an objective that measures the performance of the model.

The results of the evaluations presented showed that the transformer-based approaches were able to outperform the CRF-based solution, while reducing the computational load that the proposed architecture imposes on our robotic platforms and also lowering the amount of time required to perform the entire task (prediction + synchronisation). While the results obtained show that any of the approaches tested can predict appropriate gesture types given the robot’s speech and synchronise both components, and that they are all fast enough to be used in real interactions (when deployed on hardware capable of running deep learning models), there are still aspects of this research that require further development. Particularly, the main limitation of the proposed work is that the evaluation of the models is based on objective metrics exclusively. This should be complemented in future works with a user study that shows if the use of the proposed architecture enhances the way in which users perceive our robots.

## Figures and Tables

**Table 1:** Ranges for the hyperparameters optimised using Optuna. Empty cells correspond to parameters not used for training that particular approach.

	CRF-Based		Transformer-Based	
	Min	Max	Min	Max
learning rate	$1\times 10^{-3}$	$1\times 10^{-1}$	$1\times 10^{-6}$	$1\times 10^{-3}$
weight decay			$1\times 10^{-3}$	$1\times 10^{-1}$
batch size	16	128	16	128
Embedding layer size	20	400		
Hidden layer size	20	400		
Number of recurrent layers	1	6		
Dropout	0.0	0.8		
Gradient norm	1.0	10.0		
Patience	1	20		
Epochs for training	50	100		

**Table 2:** Hyperparameters used for training the different models. Empty cells correspond to parameters not used for training that particular approach.

	CRF-Based		Transformer-Based		
	Intention	Gesture	BERT	DistilBERT	RoBERTa
learning rate	0.005982	0.003296	$1.55535\times 10^{-5}$	$5.89857\times 10^{-5}$	$6.35378\times 10^{-5}$
weight decay	0.0	0.0	0.01978	0.00497	0.00271
batch size	16	2	16	32	32
Embedding layer size	162	383			
Hidden layer size	183	130			
N. of recurrent layers	2	3			
Dropout	0.36827	0.477513			
Gradient norm	7.75628	6.89757			
Patience	16	6			
Epochs for training	88	93			

**Table 3:** Results obtained from the training of the models. The highest value for each metric has been highlighted in bold.

Model	Precision	Accuracy	Recall	F-Score
CRF-based	0.7518	0.7395	0.7466	0.744
BERT	0.7772	0.7706	0.7874	0.7729
DistilBERT	0.7938	0.7827	0.7922	0.7804
RoBERTa	0.797	0.7395	0.7856	0.7801

**Table 4:** Resource usage for all four models. The GPU utilisation and memory refer to the models when deployed on the external server, while the CPU utilisation and RAM refer to the models when deployed locally. The number in each cell represents the average value, plus or minus the standard deviation.

Model	Remote		Local	
	GPU Usage (%)	GPU Memory (%)	CPU Usage (%)	RAM (%)
**BERT**	2.1951±1.6313	6.001±0.21614	205.671±62.976	6.915±0.001
**DistilBERT**	1.1463±0.9633	5.2531±0.259	148.278±48.561	5.542±0.001
**RoBERTa**	2.0244±0.908	6.2392±0.2676	222.916±70.076	7.421±0.002
**CRF-Based**	23.1±9.1164	9.1094±0.1709	560.505±51.261	8.79±0.059

**Figure A1:**
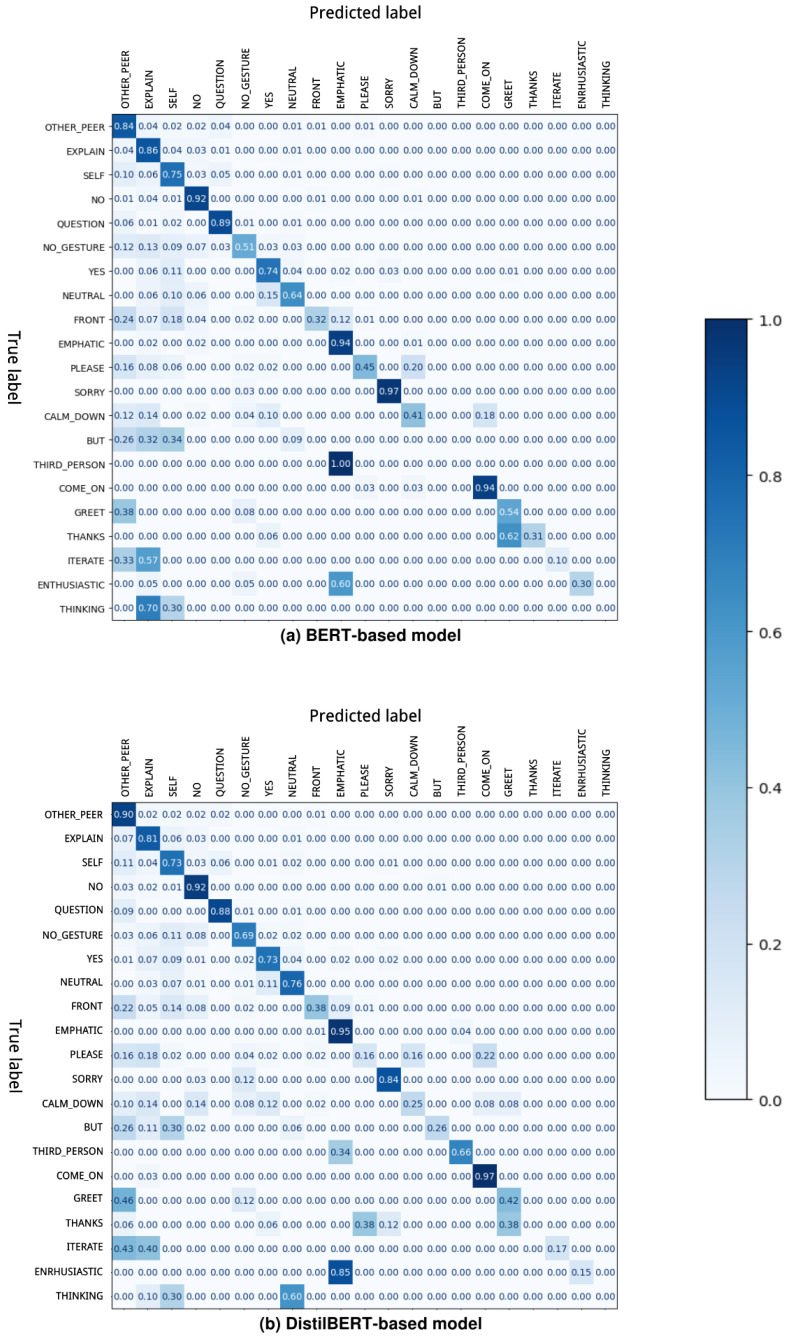
Confusion matrices showing the performance per label of the BERT-based and DistilBERT-based models.

**Figure A2:**
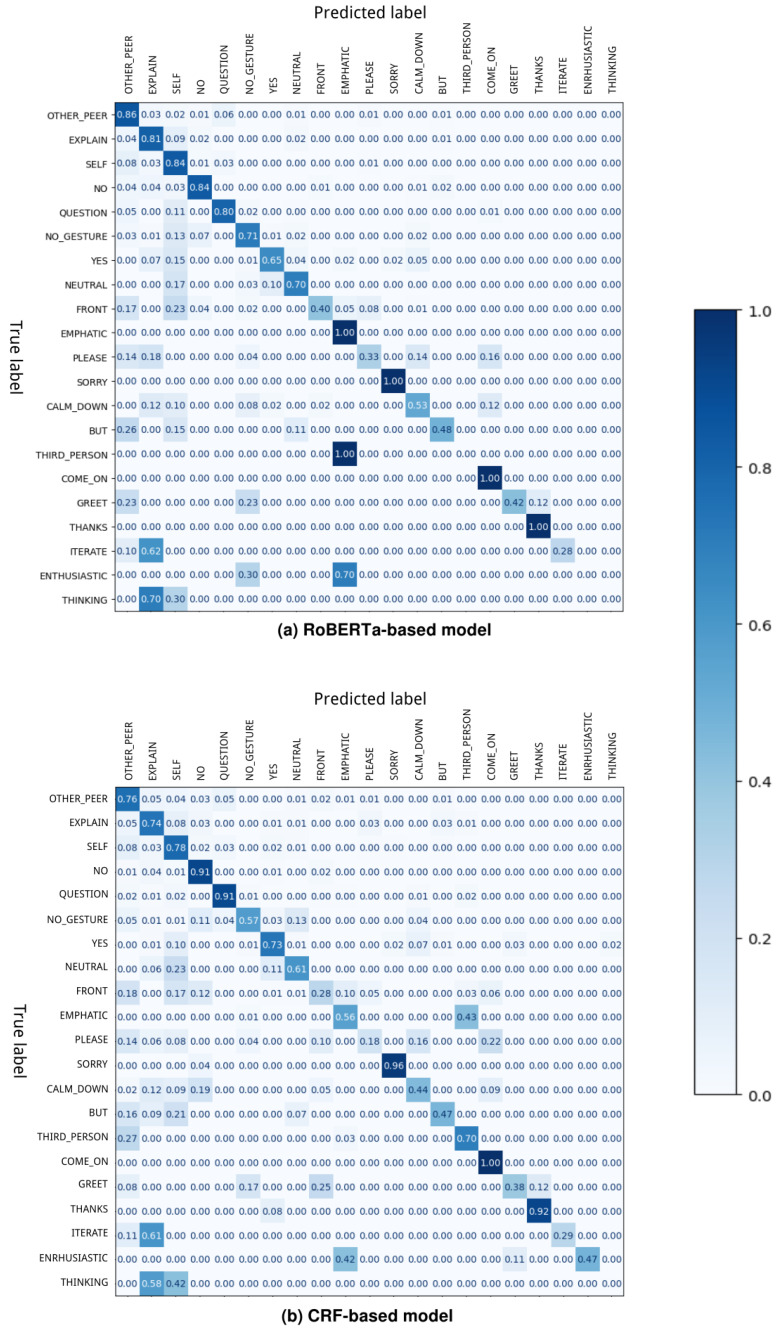
Confusion matrices showing the performance per label of the RoBERTa-based and CRF-based models.

## Data Availability

The models fine-tuned for this work are publicly available. The dataset developed for this work is publicly available.

## Appendix A. List of Communicative Intentions and Gesture Types Used in the Dataset

### Appendix A.1. Communicative Intentions

**Table A1:** Labels depicting the possible communicative intentions that the robot might use.

Label	Description
State Robot Fact	The robot presents information about itself.
Request Personal Info	Asking information about the user.
Explain	Generic utterance that serves for exposition purposes.
Ask Question	Generic question that is not about any personal information.
State Third Party Fact	The robot presents facts about a third party not present.
State User Fact	Statement that presents a fact about the user.
Voice Opinion	The robot expresses an opinion that is not about the user.
Request Action	The robot asks or demands that the user does something.
Agree	The robot is agreeing with the user’s previous statement.
Ask For Opinion	The robot asks the user to provide their opinion about something.
Refuse	The robot answers negatively, or rejects a previous statement.
Emphasize	The robot provides information on something it feels strongly about.
Notify Action	The robot tells the user about an action that it will carry on.
Counter Opinion	Provides a counter-idea to the one presented right before.
Apologize	Utterance for showing remorse (not necessarily an explicit apology).
Express Doubt	The robot provides a fact about which it is not completely sure.
Encourage	The robot aims at encouraging the user to do something.
Greet	Utterance used to initiate or end an interaction.
Confront	The robot is engaged in a heated argument with the user.
Show Empathy	The robot shows empathy towards the user’s last statement.
Thank User	Utterance used to show gratefulness towards the user.
Calm User	The robot tries to calm the user.
Make Offer	The robot offers to do something for the user.
Express Need	Utterance used to express a need so the user can act on it.
Express Enthusiasm	The robot states a fact about which it feels strongly positive.
Offer Choice	The robot offers the user a choice among multiple options.
Give Evaluation	The robot conveys an opinion about the user.

### Appendix A.2. Gesture Types

**Table A2:** Labels depicting the possible semantic values that can be conveyed with the robot’s gestures.

Label	Description
Other Peer	Extending the arm towards the other peer with the back of the hand facing them.
Explain	Gesture designed for generic situations.
Self	The speakers points towards themself with the palm of the hand.
No	Head shake to convey negation.
Question	Gesture or posture adopted for asking a generic question.
No Gesture	No action should be performed.
Yes	Head nod to express an agreement.
Neutral	Gesture that expresses doubt, usually by shrugging the shoulders.
Front	Pointing gesture with an extended finger towards the other peer.
**Emphatic**	Variation of the explain gesture with a higher intensity.
Please	Posture or gesture adopted for requesting help to the other peer.
Sorry	Posture or gesture to show remorse.
Calm Down	Gesture used to induce calm in the other peer.
But	Change in stance for punctuating a counter-idea to what has been said right before.
Third Person	Arm extended perpendicularly to the other peer, referencing an external party.
Come On	Gesture that encourages the user to do something.
Greet	A gesture used to start or finish a conversation.
Thanks	A gesture used to show gratefulness.
Iterate	Rhythmic beats used to punctuate the items in an enumeration.
Enthusiastic	Display of strong positive affect towards the content of the utterance.
Thinking	Represents the process of thinking about the information being conveyed.

## Appendix B. Example of an Instance from the Dataset Used to Train the CRF-Based Model

<?**xml** **version**="1.0" encoding="UTF-8"?> <example id="1573945815700"> <sentence>Well, for starters, what do you do?</sentence> <tokens> <token id="1" lemma="well" pos="INTJ" surface="Well" /> <token id="2" lemma="," pos="PUNCT" surface="," /> <token id="3" lemma="for" pos="ADP" surface="for" /> <token id="4" lemma="starter" pos="NOUN" surface="starters" /> <token id="5" lemma="," pos="PUNCT" surface="," /> <token id="6" lemma="what" pos="PRON" surface="what" /> <token id="7" lemma="do" pos="VERB" surface="do" /> <token id="8" lemma="-PRON-" pos="PRON" surface="you" /> <token id="9" lemma="do" pos="VERB" surface="do" /> <token id="10" lemma="?" pos="PUNCT" surface="?" /> </tokens> <intentions> <token id="1" value="B-REQUEST_PERSONAL_INFO" /> <token id="2" value="I-REQUEST_PERSONAL_INFO" /> <token id="3" value="I-REQUEST_PERSONAL_INFO" /> <token id="4" value="I-REQUEST_PERSONAL_INFO" /> <token id="5" value="I-REQUEST_PERSONAL_INFO" /> <token id="6" value="I-REQUEST_PERSONAL_INFO" /> <token id="7" value="I-REQUEST_PERSONAL_INFO" /> <token id="8" value="I-REQUEST_PERSONAL_INFO" /> <token id="9" value="I-REQUEST_PERSONAL_INFO" /> <token id="10" value="I-REQUEST_PERSONAL_INFO" /> </intentions> <gestures> <token id="1" value="B-NEUTRAL" /> <token id="2" value="I-NEUTRAL" /> <token id="3" value="I-NEUTRAL" /> <token id="4" value="I-NEUTRAL" /> <token id="5" value="I-NEUTRAL" /> <token id="6" value="B-OTHER_PEER" /> <token id="7" value="I-OTHER_PEER" /> <token id="8" value="I-OTHER_PEER" /> <token id="9" value="I-OTHER_PEER" /> <token id="10" value="I-OTHER_PEER" /> </gestures> </example>
